# A Multilevel Regression Model for Geographical Studies in Sets of Non-Adjacent Cities

**DOI:** 10.1371/journal.pone.0133649

**Published:** 2015-08-26

**Authors:** Marc Marí-Dell’Olmo, Miguel Ángel Martínez-Beneito

**Affiliations:** 1 CIBER Epidemiología y Salud Pública (CIBERESP), Madrid, Spain; 2 Agència de Salut Pública de Barcelona, Barcelona, Spain; 3 Institut d'Investigació Biomèdica (IIB Sant Pau), Barcelona, Spain; 4 Fundación para el fomento de la investigación sanitaria y biomédica de la Comunidad Valenciana (FISABIO), Valencia, Spain; Oswaldo Cruz Foundation, BRAZIL

## Abstract

In recent years, small-area-based ecological regression analyses have been published that study the association between a health outcome and a covariate in several cities. These analyses have usually been performed independently for each city and have therefore yielded unrelated estimates for the cities considered, even though the same process has been studied in all of them. In this study, we propose a joint ecological regression model for multiple cities that accounts for spatial structure both within and between cities and explore the advantages of this model. The proposed model merges both disease mapping and geostatistical ideas. Our proposal is compared with two alternatives, one that models the association for each city as fixed effects and another that treats them as independent and identically distributed random effects. The proposed model allows us to estimate the association (and assess its significance) at locations with no available data. Our proposal is illustrated by an example of the association between unemployment (as a deprivation surrogate) and lung cancer mortality among men in 31 Spanish cities. In this example, the associations found were far more accurate for the proposed model than those from the fixed effects model. Our main conclusion is that ecological regression analyses can be markedly improved by performing joint analyses at several locations that share information among them. This finding should be taken into consideration in the design of future epidemiological studies.

## Introduction

In the last decade, the use of ecological regression studies taking small areas as units of study has become very popular. The objective of this type of study is to examine, from a geographical point of view, the relationship between a health-related outcome and exposure factors [1]. When small areas are used in this type of study, it is common to incorporate ideas and tools from disease mapping models, which are specifically constructed to deal with these units of study. One of the most popular disease mapping proposals is the Bayesian hierarchical model by Besag, York and Mollié [2], hereafter referred to as BYM. The main feature of this model is the incorporation of two random effects to explain geographical variability among units, one allowing for spatial dependence and another allowing for independence between them. The BYM approach mixes these two components as a function of their weight in the observed data.

Recently, a growing number of studies have been published on socioeconomic inequalities in mortality at the small-area level in non-adjacent cities (or metropolitan areas) (for example, see [3–9]). Briefly, by means of small-area ecological regression models, these studies assessed the association between mortality and economic deprivation. However, separate regression models were fitted for each city, so that city-specific association estimates were derived with no further use beyond the cities in the study.

To our knowledge, only one study, by Martinez-Beneito et al., has conducted a joint analysis of all cities considered [10]. That meta-analysis was carried out in a previous city-independent analysis to obtain a general overview of socioeconomic inequalities across several cities. In contrast to previous studies, the results of the latter (joint) analysis can be extrapolated to other similar cities not included in the original study, since the available cities were treated as a sample of large cities from the region/country of interest, and thus the results could be generalised to the entire region/country. However, as previously mentioned, the relative risk (RR) estimates studied by Martinez-Beneito et al. [10] were derived from a previous analysis, which considered them as completely independent quantities. That meta-analysis performed a post-analysis of the previous independent estimates, whereas it seems much more appropriate to estimate all of them in a single joint analysis. Such an analysis would allow incorporation of the variability in risks both within and between cities and, if necessary, would simultaneously allow incorporation of the dependence of these two sources of variability.

Some of the previously-mentioned studies found a spatial pattern, at the inter-city level, of socioeconomic inequalities in mortality, suggesting spatial dependence between cities [4,6,8]. This dependence is portrayed descriptively because it cannot be quantified objectively. For example, Borrell et al. [4] analysed inequalities in total mortality in 16 European cities and found that relative inequalities were greater in eastern and northern European cities and were smaller in some western (in men) and southern (in women) European cities. This pattern had been observed at the country-level and has both conceptual and methodological explanations [11]. However, by analysing cities independently, spatial dependence between cities has been ignored, even though, as already shown, the nature of the data makes it very likely that a spatial structure can be found within them.

In this study, we propose a multilevel ecological regression model in which the first level of the analysis consists of small areas nested within cities and the second level corresponds to the cities themselves. This analysis allows the identification of possible spatial structures at both of the levels considered. This proposal is illustrated by an example from real data exploring the association between unemployment and lung cancer mortality among men in 31 Spanish cities from 2002 to 2007.

## Methods

### Statistical modelling

We propose three different Bayesian hierarchical models for performing multilevel ecological regression, all three corresponding to different assumptions. For all proposals, *O*_*ij*_ denotes the number of observed cases in census tract *i* (*i* = 1,…,*n*_*j*_) of city *j*, the administrative divisions used from now on to introduce the models below. Similarly, *E*_*ij*_ denotes the expected number of cases, *θ*_*ij*_ the relative risk, and *X*_*ij*_ the unemployment rate of the corresponding census tract.

#### Model 1 (M1)

As usual in disease mapping studies, we account for the spatial structure of the relative risks *θ*_*ij*_ within each city. To do this, we consider two random effects, as proposed by Besag, York and Mollié (BYM). The spatial random effects for each city *j* (*S*_*ij*_) are assumed to follow an intrinsic conditional auto-regressive distribution (ICAR) [2] with different variances for each city, $\sigma_{S}^{2}{}_{j}$. Similarly, the heterogeneous (non-spatial) random effects (*H*_*ij*_) are assumed for each city to follow independent normal distributions with mean zero and variance, $\sigma_{H}^{2}{}_{j}$. Uniform prior distributions between 0 and a vague upper limit were assigned to the standard deviations (*σ*_*S j*_ and *σ*_*H j*_) of the random effects [12]. Note that independent variances have been assumed for each of the cities instead of a common variance for all of them. Thus, each city can show a distinct degree of spatial dependence in their relative risks.

In addition to these census-tract-level random effects, each city also has a linear component that depends on the covariate of interest, so that the relationship between this factor and the risks can be explored. We assumed the effect of both the intercept and the covariate to be completely different and independent for each city. Thus, M1 can be formulated as follows:

$$\begin{array}{c} O_{ij}\sim Poisson\left(E_{ij}\theta_{ij}\right),\;i=1,\ldots ,n_{j},\;j=1,\ldots ,J\\ \log\left(\theta_{ij}\right)=b_{1j}+b_{2j}\cdot X_{ij}+S_{ij}+H_{ij}\\ b_{1j},b_{2j}\sim Uniform\left(-\infty ,+\infty \right)\\ S_{ij}\sim Intrinsic-CAR\left(\sigma_{S}^{2}{}_{j}\right)\\ H_{ij}\sim Normal\left(0,\sigma_{H}^{2}{}_{j}\right)\\ \sigma_{S}{}_{j},\sigma_{H}{}_{j}\sim Uniform\left(0,l\right) \end{array}$$

For *l* being a suitable upper limit, making the corresponding uniform distributions vague. This model is equivalent to performing separate ecological regressions (with BYM random effects) for each city which, as mentioned in the Introduction, is the most common procedure when analysing multiple cities in a single study. This model and those that follow will return the RR per 1% increase in the unemployment rate for each city *j* (exp(*b*_2*j*_)) as the main outcome that summarises the relationship between the covariate and the observed counts under study.

#### Model 2 (M2)

This model is based on M1, but now the intercepts *b*_1*j*_ and the coefficients associated with the covariate *b*_2*j*_ are considered to be random effects rather than fixed effects for each city (Model 1). Specifically, both *b*_1*j*_ and *b*_2*j*_ are assumed to follow normal distributions with means and variances that are unknown but common to all the cities. In contrast to M1, information on the values of *b*_1*j*_ and *b*_2*j*_ for j = 1,…,31 will now be shared between cities through their standard deviations. The elements of these two vectors are not independent, as in M1, but are conditionally independent, given $\sigma_{b_{1}}^{2}$ and $\sigma_{b_{2}}^{2}$, respectively. Thus, they are sharing their information. Model 2 can be formulated as follows:

$$\begin{array}{c} O_{ij}\sim Poisson\left(E_{ij}\theta_{ij}\right),\;i=1,\ldots ,n_{j},\;j=1,\ldots ,31\\ \log\left(\theta_{ij}\right)=b_{1j}+b_{2j}\cdot X_{ij}+S_{ij}+H_{ij}\\ b_{1j}\sim Normal_{J}\left(\alpha ,\sigma_{b_{1}}^{2}\right)\\ b_{2j}\sim Normal_{J}\left(\beta ,\sigma_{b_{2}}^{2}\right)\\ S_{ij}\sim Intrinsic-CAR\left(\sigma_{S}^{2}{}_{j}\right)\\ H_{ij}\sim Normal\left(0,\sigma_{H}^{2}{}_{j}\right)\\ \alpha ,\beta \sim Uniform\left(-\infty ,+\infty \right)\\ \sigma_{b_{1}},\sigma_{b_{2}},\sigma_{S}{}_{j},\sigma_{H}{}_{j}\sim Uniform\left(0,l\right) \end{array}$$

In addition to the RR of death for each city (exp(*b*_2*j*_)), this model estimates an overall pooled RR for all cities (exp(*β*)), or, more precisely, for the set of cities from which the available cities are drawn. This common RR cannot be estimated in M1 because the RR in that model are considered as fully independent between cities and therefore it would make no sense to derive any joint estimate in M1.

#### Model 3 (M3)

Up to this point, M1 and M2 have only considered spatial dependence within cities (at census tract level), now M3 also accounts for spatial dependence between cities using a geostatistical approach [13]. Specifically, this new model is similar to M2 but considers that the geographic arrangement of cities could induce spatial dependence between their RR. M3 accounts for this spatial dependence by assuming multivariate normal prior distributions for both *b*_1*j*_ and *b*_2*j*_ and modelling their covariance matrices. Specifically, these matrices are modelled as an exponential function that depends inversely on the distance between cities, so that the covariance is high for geographically close cities and tends to disappear with increasing distance. Thus, M3 can be formulated as:

$$\begin{array}{c} O_{ij}\sim Poisson\left(E_{ij}\theta_{ij}\right),\;i=1,\ldots ,n_{j},\;j=1,\ldots ,31\\ \log\left(\theta_{ij}\right)=b_{1j}+b_{2j}\cdot X_{ij}+S_{ij}+H_{ij}\\ b_{1j}\sim Normal_{J}\left(\alpha ,\Sigma_{1}\right)\\ b_{2j}\sim Normal_{J}\left(\beta ,\Sigma_{2}\right)\\ \Sigma_{1}=\sigma_{1}^{2}\cdot \exp\left(-\phi_{1}\cdot D\right)\\ \Sigma_{2}=\sigma_{2}^{2}\cdot \exp\left(-\phi_{2}\cdot D\right)\\ S_{ij}\sim Intrinsic-CAR\left(\sigma_{S}^{2}{}_{j}\right)\\ H_{ij}\sim Normal\left(0,\sigma_{H}^{2}{}_{j}\right)\\ \alpha ,\beta \sim Uniform\left(-\infty ,+\infty \right)\\ \sigma_{S}{}_{j},\sigma_{H}{}_{j},\sigma_{1},\sigma_{2}\sim Uniform\left(0,l\right)\\ \phi_{1},\phi_{2}\sim Uniform\left(a,b\right) \end{array}$$

where the extremes a and b of the uniform distribution of *ϕ*_1_ and *ϕ*_2_ are selected so that the covariance for any pair of cities does not take excessively low or high values that may induce numerical problems, as suggested by Wang et al. [14].

The covariance matrix of *b*_2*j*_ depends on *σ*_2_, the standard deviation controlling the magnitude of these values and a correlation matrix (similarly for *b*_1*j*_). This matrix depends on D, the matrix of distances between cities and *ϕ*_2_, which controls the decay of spatial dependence as a function of the distance between cities. Within this model, we can also calculate *R* = −ln(0.05)/*ϕ*_2_, usually known in geostatistics as the effective range [15], which is interpreted as the distance at which the spatial dependence between cities becomes negligible. This is also an interesting outcome of M3, in that it summarizes the spatial pattern observed. Finally, exp(*β*) can be interpreted as an overall pooled RR for the whole study, as in M2.

All models were fitted using a fully Bayesian approach. Posterior distributions were obtained by means of Monte Carlo methods based on Markov chains using the WinBUGS program (version 1.4.3) [16] executed from R (version 3.1.2) [17] through the R library R2WinBUGS (version 2.1–19) [18]. Seven Markov chains were run (in parallel) with 100,000 iterations, of which the first 10,000 were rejected as burn-in, in order to ensure the convergence of the parameters in the model. Of these, only 1 out of every 126 iterations was retained, with the aim of reducing the storage requirements of the simulation process. Convergence was assessed using the Brooks-Gelman-Rubin statistic (R-hat) and the effective sample size of the chains (n.eff). Specifically, we required the statistics R-hat<1.1 and n.eff >100 for each parameter in the model [19]. We also performed a visual check of the chains for a sample of the saved variables but not a visual check of all the saved variables, which was precluded by the high number of variables (around 2000 for each model). Posterior means were used as point estimates of the parameters in the model. Some of these estimates are accompanied by their corresponding 95% credible intervals (95%CI).

One of the main advantages of M3 is that, by integrating a geostatistical approach, it allows us to predict the RRs in locations where there are no available city data. Thus, the RR can be extrapolated to places where they cannot be directly observed, simply by the proximity of each of these points to the cities for which information is available. Specifically, the RR can be estimated for any location in the region of the study if there is a city of the same kind as those considered in the analysis. That is, if the available cities were, for example, a sample of the large cities in a country, the predictions made would correspond to the RRs that could be expected at any geographical location if a large city were located there. This will make these spatial predictions comparable since they correspond to similar units instead of reproducing the demographic pattern of the region of study. To this end, we used Kriging Bayesian spatial prediction as specified in part 4 of Diggle et al. [13]. Further details of this procedure are provided in S1 Appendix.

We used the deviance information criterion (DIC) [20] as a model selection criterion for our analysis. Models with smaller DIC are preferred over models with larger DIC.

### A lung cancer mortality study in Spain

**Fig 1:**
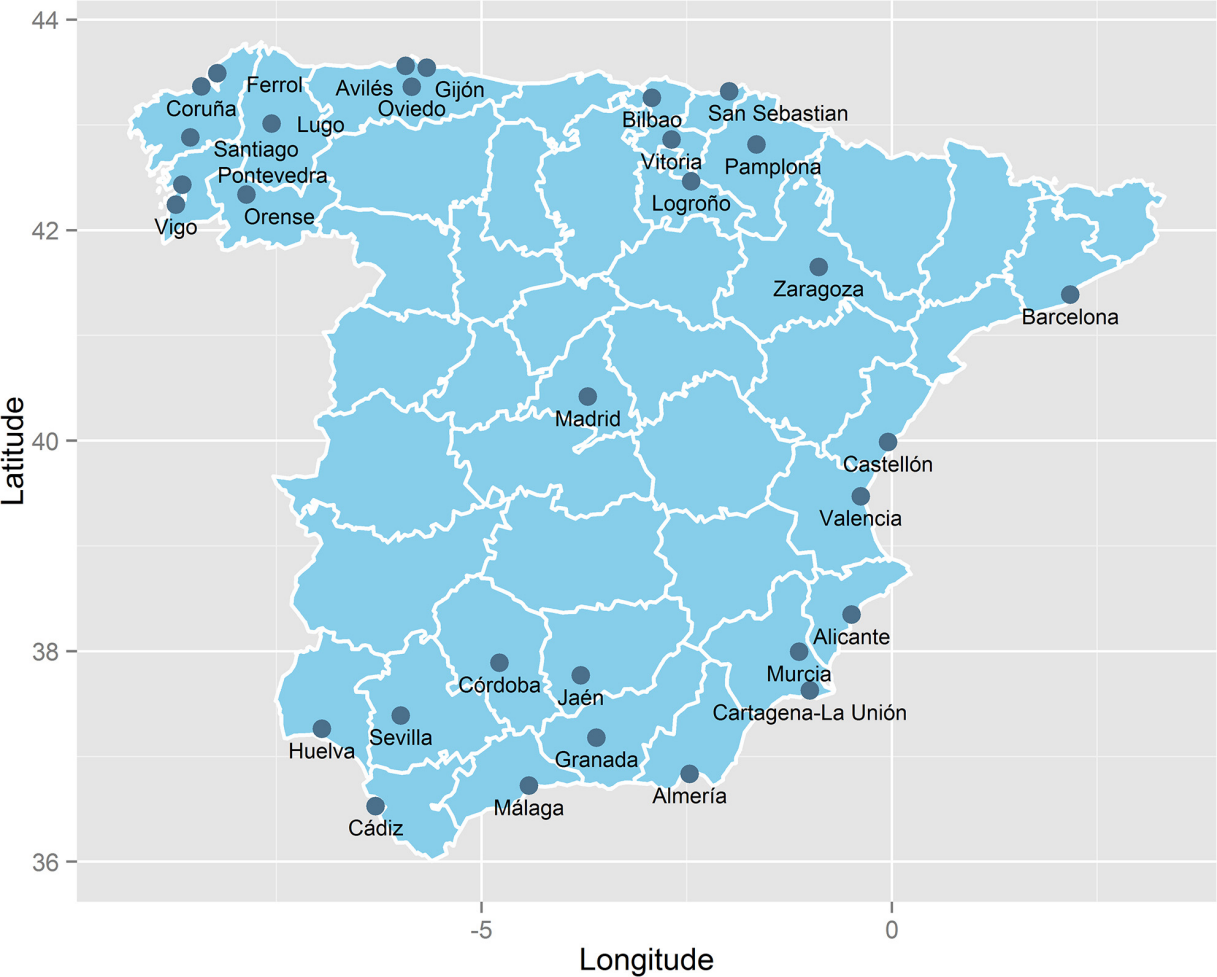
Geographic distribution of the Spanish cities studied.

The proposed models will be illustrated and assessed with a study conducted with real data. This was a cross-sectional ecological study forming part of the MEDEA project (http://www.proyectomedea.org/). The units of analysis were the census tracts of 31 Spanish cities (Fig 1), as defined in the 2001 Spanish Population and Housing Census. The study population consisted of men residing in the 31 cities during the period 2002–2007

These 31 cities accounted for 27.4% of the Spanish population in 2007 and are spread throughout mainland Spain (Fig 1). They include the 10 most populated cities in Spain and all of them are within the 80 most populated Spanish cities. Therefore, they represent a sample of the most populated Spanish cities.

In this study, we analysed male deaths due to malignant neoplasm of the trachea, bronchus or lung (henceforth ‘lung cancer’) during the period 2002–2007. Specifically, we selected deaths classified in codes C33 and C34 of the International Classification of Diseases, 10^th^ revision (ICD-10). Mortality data were obtained from the mortality registers of the corresponding autonomous communities. Population data were obtained either from the register of inhabitants for each city or from the National Institute of Statistics. Finally, as a covariate, we included the 2001 unemployment rate, defined as the percentage of unemployed workers in the total labour force. This socioeconomic indicator was obtained from the 2001 Spanish Population and Housing Census. Unemployment was used as a surrogate of material deprivation, which has been previously claimed to be associated with lung cancer mortality [7]. Although more elaborate deprivation indexes could have been employed for our illustration, the use of an optimal deprivation indicator is neither within the scope of this paper nor of this particular example. All data were available at census tract level for each city.

The expected numbers of cases per census tract to be used in the above-mentioned models were calculated by indirect standardization, taking the age-specific (quinquennial groups) male lung cancer mortality rates for the full set of cities as the reference.

For M3, we estimated the posterior mean of the RR, the posterior standard deviations of the RR and the probability of the RRs being greater than 1 in each of the 5,221 locations on a grid of points that we considered throughout mainland Spain. These estimates were calculated following the geostatistical approach mentioned above.

## Results and Discussion

First, after running all three models for the lung cancer dataset, model selection was performed using the DIC statistic, which indicated M3 as the model showing the best fit (DIC_M1_ = 36408.6, DIC_M2_ = 36392.1, DIC_M3_ = 36389.0). According to Spiegelhalter et al. [20], differences between M2 and M3 (their DICs differ between 3 and 7 units) are moderate, although both models are clearly better (their DICs are more than 7 units higher) to M1 in terms of DIC.

**Fig 2:**
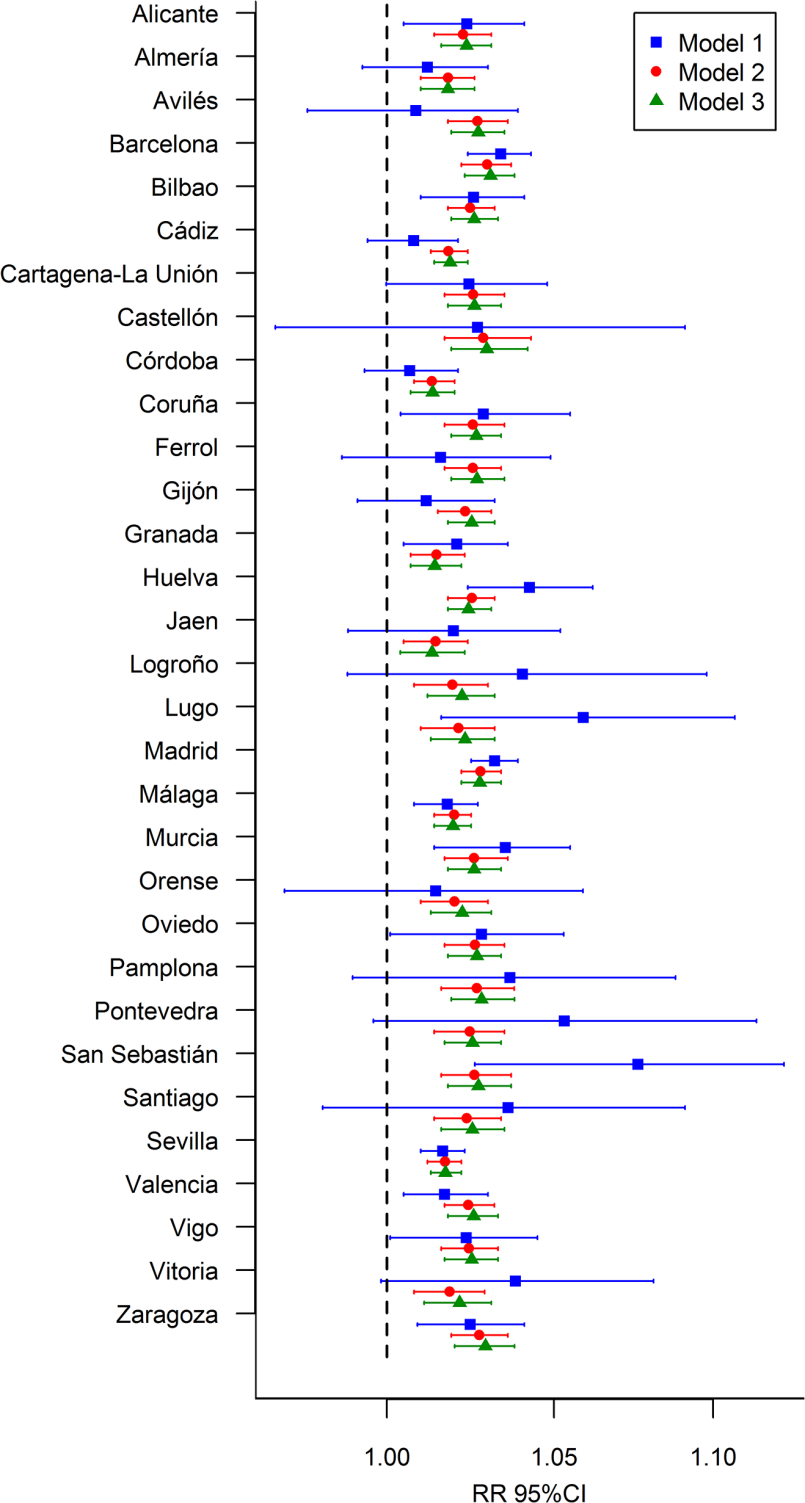
Comparative model with respect to city specific relative risks (RR) between mortality and unemployment and their 95% credible intervals (95%CI), 2002–2007.

Comparison of the RRs for all cities (Fig 2) revealed that those for M2 and M3 are much more stable than those for M1. First, the posterior means of the RRs for different cities were more similar for M2 and M3 and, second, their 95% CIs were substantially narrower. For example, for the city of Santiago, we obtained the following RRs (95% CIs) for each model: RR_M1,_ 1.036 (0.982–1.091); RR_M2,_ 1.024 (1.014–1.034); RR_M3_, 1.025 (1.016–1.035). The widths of the CIs for M2 and M3 are reduced by ≥75% with respect to those of M1. Consequently, the use of one model or another can lead to very different conclusions.

As previously mentioned, it is common to analyse each city separately and thus to report the results of M1, which show more variability. In our opinion, the joint analysis of all cities using M2 and M3 is more appropriate, since the use of random effects allows information to be shared between cities, taking into account that they are not isolated entities. Although M2 and M3 estimated similar RRs in our particular study, M3 may sometimes have particular advantages. For example, for cities with few small areas, the uncertainty of the posterior distribution of the RR will be very high (resulting in a wide 95% CI). In this case, both M2 and M3 will markedly improve the RR estimate, although, if there is a spatial pattern in the RRs, M3 will take advantage of it, returning more accurate estimates. Moreover, if the data show weak spatial dependence, M3 will be able to reproduce that kind of pattern by yielding a short effective range and therefore the use of a spatial model in that case should not be a problem.

**Fig 3:**
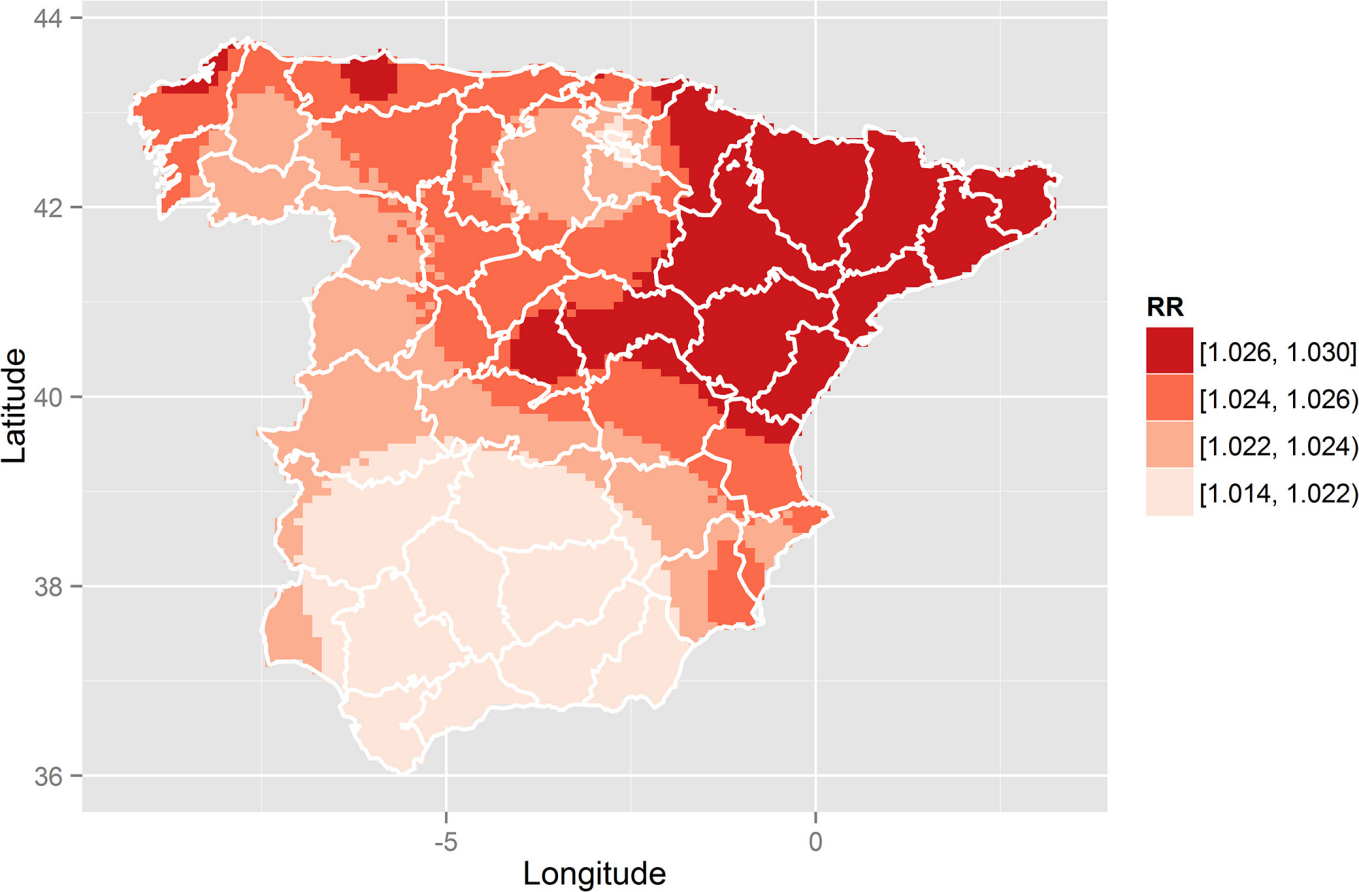
Predicted relative risks (RR) between lung cancer mortality in men and unemployment throughout peninsular Spain, 2002–2007.

As described in the previous section, M3 may use (Bayesian) Kriging to predict the RR at locations where there is no available information. Fig 3 shows a map of the predicted RRs on a grid of 5221 points throughout the whole mainland of Spain. Grid points are horizontally spaced about 11.5 kilometres apart and are vertically spaced about 8.7 kilometres apart. This figure shows a clear northeast-southwest spatial pattern for the RRs, decreasing toward the southwest. This spatial pattern is confirmed by the value of the effective range, which has a posterior mean of 426 kilometres. That is, at least 426 kilometers is the distance that must be covered to find two cities with virtually independent RR estimates (note that the longest straight-line distance within peninsular Spain is 1079 kilometres). As mentioned in the introduction, various European studies analysing data from different countries [11] or cities [4] have suggested the existence of a spatial pattern in socioeconomic inequalities for certain causes of death. However, none of these studies have quantified or shown this pattern and their conclusions were based only on the visual interpretation of their results. M3 is able to estimate this spatial pattern and even to quantify its ‘spatiality’ in terms of its effective range, allowing comparison of different patterns corresponding to different causes of mortality. Moreover, M3 also estimates the spatial standard deviation, *σ*_2_, which quantifies the variability of RR between cities. Therefore, M3 provides two parameters (the effective range and the spatial standard deviation), allowing objective comparison of any two geographical patterns.

**Fig 4:**
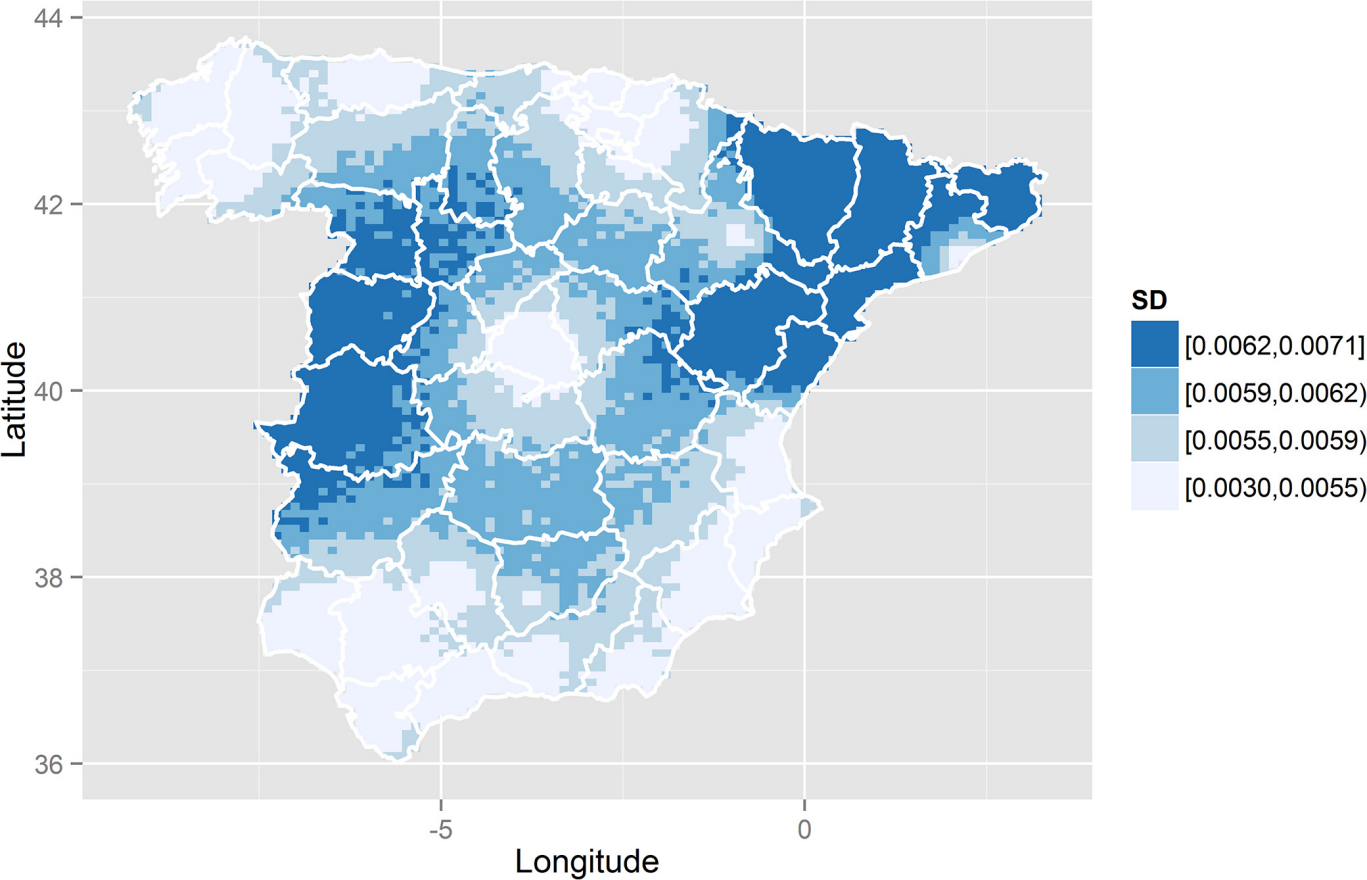
Posterior standard deviations (SD) of the predicted relative risks between lung cancer mortality in men and unemployment throughout peninsular Spain, 2002–2007.

Estimating the full posterior distributions of the predicted RRs allows visualization of other estimates beyond their posterior means, as in Fig 3. Thus, for each RR, the posterior probability of being greater than 1 could be plotted, in a similar manner to that shown Fig 3. In our study, this plot is not shown since the association found is very strong for the whole region of the study, making those probabilities higher than 0.9 all around, and thus producing a completely flat map. Nevertheless, this is not always the case, especially in studies without a clear association between the covariate and the cause of mortality. In these cases, it would be possible to distinguish between areas with significant association and those without and, thus, to identify which of the risks shown in Fig 3 are ‘significantly higher than 1’ and which are not. Instead of the map with the above-mentioned probabilities, we plotted a map with the posterior standard deviation of the RRs at each point in the grid considered (Fig 4). In this map, darker regions correspond to regions with larger posterior standard deviations (those with least accurate RRs estimates). Therefore, dark values are mainly observed in the regions furthest away from any of the cities in the study.

Finally, M2 and M3 also yield overall RRs (oRR) for the cities as a whole. The posterior means of the oRR for these two models are oRR_M2_ = 1.023 (95%CI = 1.019–1.028) and oRR_M3_ = 1.025 (95%CI = 1.019–1.031). Both models provided very similar posterior means for oRR, although the posterior variability of M3 was greater, a result of considering correlation in the data (dependence makes estimates less accurate). Therefore, considering the data as spatially independent, when they are not, would lead to erroneously optimistic estimates (in terms of their variability). Moreover, if M3 estimated very little variability between cities (*σ*_2_ ≈ 0), it would make particular sense to estimate an overall RR for the cities as a whole, instead of independent (and noisy) estimates of this quantity for each city.

## Conclusions

In this study we propose a new ecological regression model for studies in small areas of non-adjacent cities. This model merges a frequently used proposal in disease mapping, the BYM model, with Bayesian geostatistical models [21], both of which are jointly integrated in a multilevel approach.

For this kind of multicentre study, it is common to perform independent analyses for each node, in our case cities, and to subsequently merge their results. In this paper, we show that this procedure overestimates the uncertainty of RRs in comparison to models that jointly analyse all cities, sharing their information by means of random effects (M2 and M3). Furthermore, the fixed effects modelling of M1 makes their estimates only applicable to the set of cities considered in the study, that is, their results cannot be extrapolated to any other city, which limits the interest of the results. In this regard, the results from M2 and M3 are much more useful and general.

In our illustration, although the results for M2 and M3 were similar in terms of the estimated RR for each city, in our opinion, M3 shows at least three clear advantages over M2. First, it allows us to account for the spatial dependence both within and between cities. Second, it allows us to objectively detect and quantify (using the effective range) the spatial structure in the data. Finally, RRs (and even their significance) can also be predicted at locations with no available data. In our opinion, because of these advantages and the ability of M3 to adapt to patterns showing different degrees of spatiality (even spatial independence), it is the most appropriate model for this kind of study.

Finally, when studying the association between a health outcome and an exposure factor, it may be useful both to "map" at locations where information is available and to visually assess the similarity of their spatial patterns. The proposed model provides a different approach, allowing this association to be directly plotted in a continuous map. In our opinion, this improves understanding of this association. For example, as an end result, this article describes the spatial distribution of socioeconomic inequalities in mortality, allowing identification of the areas with the greatest inequalities, which are therefore the most amenable to intervention. Thus, the proposed models may be a valuable tool for policy makers to make a more appropriate investment of resources.

## Supporting Information

S1 Appendix Spatial prediction using Model 3. (DOC)
