# Supplementary material for: A Multilevel Regression Model for Geographical Studies in Sets of Non-Adjacent Cities
Source: PLoS One. 2015 Aug 26;10(8):e0133649. doi: 10.1371/journal.pone.0133649 (PMC4550405; doi:10.1371/journal.pone.0133649)
Supplement: S1 Appendix — (DOC) [file pone.0133649.s001.doc]

**S1 Appendix. Spatial prediction using Model 3**

As described in the Methods section, Model 3 returns $b_{2j}$ for each city *j* ($j=1,...,31$) at spatial locations $\mathbf{x}_{j}$. A grid of 5221 points is then defined across the entire surface of peninsular Spain, at which the value of RR is to be predicted. Let $\mathbf{x}_{k}^{*}$ be the vector of 5221 elements ($k=1,...,5221$) with spatial locations corresponding to the points of the grid. Finally, we define a new joint vector $\mathbf{x}'=\left(\mathbf{x},\mathbf{x}^{*}\right)$ with 31+5221=5252 elements. The distance matrix $D'$ of the set of spatial locations of the vector $\mathbf{x}'$ is then calculated and has a size of $5252\times5252$. Thus, following step 4 of part 4 of Diggle *et al*. [1], for each of the simulations obtained in the MCMC, we calculated $\Sigma=\sigma_{2}^{2}\cdot\exp\left(-\varphi_{2}\cdot D'\right)()$, which is a $5252\times5252$ covariance matrix that can be represented as:

$$\Sigma=\left(\begin{matrix} \begin{matrix} \sigma_{1,1} & \cdots& \sigma_{1,31} \\ \vdots& \ddots& \vdots\\ \sigma_{31,1} & \cdots& \sigma_{31,31} \end{matrix} & \begin{matrix} \sigma_{1,32} & \cdots& \sigma_{1,5252} \\ \vdots& \ddots& \vdots\\ \sigma_{31,32} & \cdots& \sigma_{31,5252} \end{matrix} \\ \begin{matrix} \sigma_{32,1} & \cdots& \sigma_{32,31} \\ \vdots& \ddots& \\ \sigma_{5252,1} & \cdots& \sigma_{5252,31} \end{matrix} & \begin{matrix} \sigma_{32,32} & \cdots& \sigma_{32,5252} \\ \vdots& \ddots& \vdots\\ \sigma_{5252,32} & \cdots& \sigma_{5252,5252} \end{matrix} \end{matrix}\right)$$

where

$\Sigma_{11}=\left(\begin{matrix} \sigma_{1,1} & \cdots& \sigma_{1,31} \\ \vdots& \ddots& \vdots\\ \sigma_{31,1} & \cdots& \sigma_{31,31} \end{matrix}\right)$,

$\Sigma_{12}=\left(\begin{matrix} \sigma_{1,32} & \cdots& \sigma_{1,5252} \\ \vdots& \ddots& \vdots\\ \sigma_{31,32} & \cdots& \sigma_{31,5252} \end{matrix}\right)$,

$\Sigma_{22}=\left(\begin{matrix} \sigma_{32,32} & \cdots& \sigma_{32,5252} \\ \vdots& \ddots& \vdots\\ \sigma_{5252,32} & \cdots& \sigma_{5252,5252} \end{matrix}\right)$,

$\mathbf{S}=\mathbf{b}_{\mathbf{2}}-\beta$,

and values of the following multivariate normal are obtained

$\mathbf{V}\sim MVN\left(\Sigma_{12}^{T}\Sigma_{11}^{-1}\mathbf{S},\Sigma_{22}-\Sigma_{12}^{T}\Sigma_{11}^{-1}\Sigma_{12}\right)$.

Finally, $\mathbf{S}^{\mathbf{*}}=\mathbf{V}+\beta$ will be a vector of 5,221 elements, and the RR for each point $\mathbf{x}_{k}^{*}$ of the grid will be obtained, and $RR_{k}=\exp\left(S_{k}^{*}\right)$ is calculated for each element $k=1,...,5221$. The posterior means of the predicted RRs are represented on a map.

**References**

1. Diggle PJ, Tawn JA, Moyeed RA. Model-based geostatistics*. J R Stat Soc Ser C (Applied St*at. Wiley Online Library; 199**8;**47(3):299–350.
